# Regulation of osmoadaptation in the moderate halophile *Halobacillus halophilus*: chloride, glutamate and switching osmolyte strategies

**DOI:** 10.1186/1746-1448-4-4

**Published:** 2008-04-28

**Authors:** Stephan H Saum, Volker Müller

**Affiliations:** 1 Molecular Microbiology & Bioenergetics, Institute of Molecular Biosciences, Goethe University Frankfurt, Frankfurt am Main, Germany

## Abstract

The moderate halophile *Halobacillus halophilus *is the paradigm for chloride dependent growth in prokaryotes. Recent experiments shed light on the molecular basis of the chloride dependence that is reviewed here. In the presence of moderate salinities *Halobacillus halophilus *mainly accumulates glutamine and glutamate to adjust turgor. The transcription of *glnA2 *(encoding a glutamine synthetase) as well as the glutamine synthetase activity were identified as chloride dependent steps. *Halobacillus halophilus *switches its osmolyte strategy and produces proline as the main compatible solute at high salinities. Furthermore, *Halobacillus halophilus *also shifts its osmolyte strategy at the transition from the exponential to the stationary phase where proline is exchanged by ectoine. Glutamate was found as a “second messenger” essential for proline production. This observation leads to a new model of sensing salinity by sensing the physico-chemical properties of different anions.

## Chloride, an abundant anion in nature

Together with flourine, bromine, iodine and astatine, chlorine belongs to the seventh group in the periodic table of elements, the "halogens". Chloride is one of the most abundant anions on earth and its content can make up to 75% in non-saline soil [1] or 90% in saline soils [2] of all inorganic anions. Huge amounts of chloride can also be found in marine habitats where its concentration can reach up to 550 mM (in combination with the corresponding cation Na^+^). This represents about 95% of the solvated anions. Due to this ubiquitous availability it is reasonable to assume that chloride is involved in biological processes.

## Chloride is essential for some extremely halophilic prokaryotes for osmoregulation

Halophilic organisms are abundant in nature and thrive in habitats where molar concentrations of salts (mainly NaCl) are present and, consequently, very low water potentials exist. However, every living cell is dependent on the availability of liquid water and therefore has to counterbalance the external osmotic conditions. To serve this purpose, and despite the different osmotic demands, only two main strategies have evolved in nature.

A very abundant strategy to counteract the loss of water under hyperosmotic conditions is the accumulation of compatible solutes [3-6]. Compatible solutes are *per definition *small, organic molecules that are soluble in water to molar concentrations and which do not interfere with cell metabolism [7]. Although a great spectrum of compatible solutes is used in the three domains of life, almost all can be assigned to two chemical classes: (i) the amino acids and their derivatives, such as glycine betaine, glutamine, glutamate, proline, ectoine or N-acetyl-β-lysine [8,3-15] and (ii) the sugars and polyols, such as trehalose, glycerol, di-*myo*-inositol-1,1'-phosphate (DIP) or sulfotrehalose [16-22]. The accumulation can be accomplished either by uptake from the medium or by *de novo *synthesis.

Contrary to the accumulation of compatible solutes, some halophilic, anaerobic Bacteria (*Halanaerobiales*) and some Archaea (*Halobacteriales*) accumulate KCl to molar concentrations to counterbalance the external salinity [23-27]. Although almost nothing is known about the mechanism of KCl accumulation in *Halanaerobiales*, the process is well understood in *Halobacteriales*. The electrical potential (Δψ) that drives the uptake of potassium ions in these organisms results from the concerted action of the membrane bound proton-pump bacteriorhodopsin and the "proton gradient-consuming" proteins ATP synthase and Na^+^/H^+ ^antiporter [28]. Potassium is taken up *via *a K^+ ^uniport mechanism. To enable such a transport the electrical potential (Δψ) has to be greater than the diffusion potential of K^+ ^(Δψ _K_). The counterion chloride is taken up either by primary or secondary transporters [29]. In the dark, a light-independent Cl^-^/Na^+ ^symporter is employed [30], but only little is known so far about this transport mechanism and the transporter is unknown. A far better understood transport of Cl^- ^is accomplished *via *the light-driven chloride pump halorhodopsin [31]. Halorhodopsin is a heptahelical membrane protein and together with bacteriorhodopsin and sensory rhodopsins belongs to the subfamily of archaeal rhodopsins [32,33]. To its only lysine residue (Lys^242^) a retinal is bound covalently as a protonated Schiff base [33]. This Schiff base, together with the surrounding amino acids, forms the active centre of the chloride pump, which is able to absorb green light with a maximal wavelength of 578 nm. The absorption of one photon triggers a photochemical reaction that leads from all-trans to a 13-cis configuration. This isomerization reaction is coupled to the transport of one molecule of Cl^- ^from the surrounding medium into the cell [34,35].

To date, only one representative of the aerobic bacteria is known that also shares the strategy of KCl accumulation to cope with extreme salinities. The extremely halophilic *Salinibacter ruber *was isolated from saltern crystallizer ponds in Spain [36] and was reported to need a minimal salt concentration of 1.7 M to allow growth. The optimal salinity was found between 2.5 and 3.9 M NaCl. It accumulates very high concentrations of Cl^- ^and K^+^/Na^+^, while other solutes were found only in minor concentrations [37]. Although biochemical analyses addressing the uptake of K^+ ^and Cl^- ^are not yet available, it was concluded from genome analyses that potassium could be taken up via a TrkHA transport mechanism. *S. ruber *possesses 4 copies of a putative *trkA *and two copies of a putative *trkH *gene that seem to originate from different sources *via *lateral gene transfer [38]. TrkH is the membrane spanning translocating subunit and TrkA is the cytoplasmic membrane surface protein that binds NADH/NAD^+ ^and which is essential for the transport activity [39]. Similar to the halobacteria, chloride could be taken up by the means of the chloride pump halorhodopsin. Altogether 4 putative genes encoding a rhodopsin were identified in the genome. Two of which were, based on sequence similarity, assigned to be sensory rhodopsins, one to be a proton pump and one a chloride pump [38]. Additionally, two copies of Na-K-Cl cotransporter – genes were identified that are common in eukaryotes but seldom found in prokaryotes. The physiological role of such a transporter is unknown for prokaryotes, but eventually contributes to the accumulation of K^+ ^and Cl^- ^in *S. ruber *cells [38].

As a prerequisite for this so called "salt-in-cytoplasm" strategy these organisms have a highly adapted, salt-tolerant or even salt-dependent enzyme equipment, that shows a high amount of acidic and a low amount of aromatic amino acid residues compared to non halophilic organisms [40,23,42]. This adaptation, however, sets clear restrictions on the habitat in which these organisms can exist, since the proteins always need a quite high intracellular salt concentration for correct protein folding and activity [24]. However, it should be mentioned that this is rather a tendency than a rule. It was reported for *Haloferax volcanii *(*Halobacteriales*), for example, that this organism is able to adapt to salinities between 1 M and almost saturation [43,44], but nothing is known about the mechanism of osmoregulation in *H. volcanii*.

## *Halobacillus halophilus *– the paradigm for chloride dependent processes in moderately halophilic bacteria

**Figure 1:**
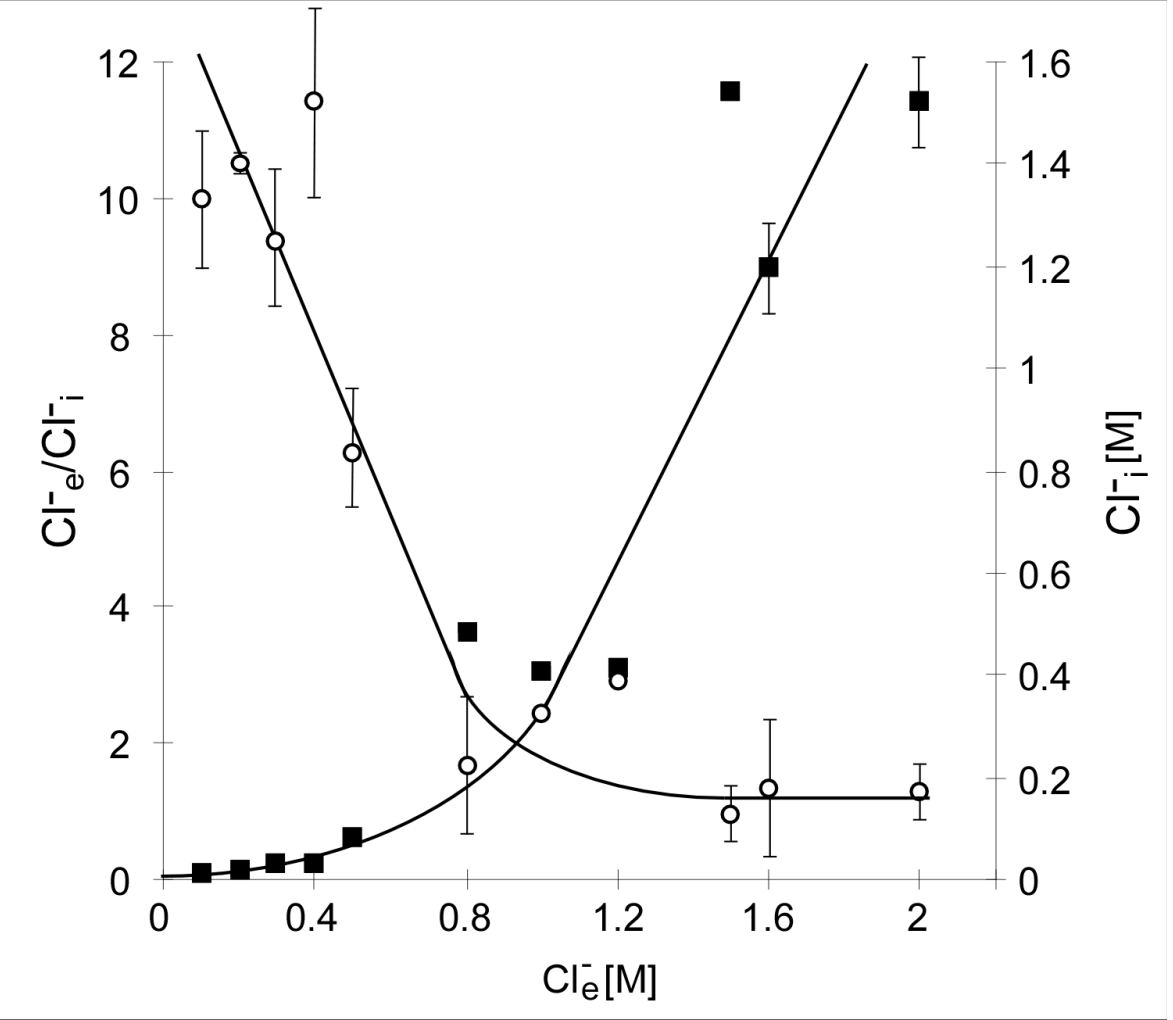
**The intracellular Cl^- ^concentrations (Cl^-^_i_) depend on the external Cl^- ^concentrations (Cl^-^_e_) in *Halobacillus halophilus***. Cl^-^_i _concentrations (filled squares) and Cl^-^_e_/Cl^-^_i _gradients (open circles) at increasing external NaCl concentrations [47].

The moderately, halophilic bacterium *Halobacillus halophilus *was originally isolated from salt-marshes at the North Sea coast of Germany. It is an aerobic, rod-shaped, motile, and endospore-forming Gram-positive bacterium of the low GC-branch that was initially classified as *Sporosarcina halophila *[45]. Based on 16S rRNA data analysis it was later reclassified as *Halobacillus halophilus *[46]. This organism has optimal growth rates between 0.5 and 2.0 M NaCl and was reported already in its first description to grow in the presence of NaCl but not Na_2_SO_4_. This first observation for a crucial role of chloride in *Halobacillus halophilus *was later confirmed by more detailed analyses [47]. It was shown that a minimal Cl^- ^concentration of 0.2 M is needed to support cell growth and that, with exception of bromide, no other halide was able to substitute chloride in this respect. After prolonged adaptation of *Halobacillus halophilus *to nitrate, it was also able to support growth, but growth rates as well as final optical densities were lower than observed for chloride. However, it is worthwhile to note that bromide as well as nitrate is of no physiological relevance, since the concentrations used in the growth experiments are not found in nature. Additionally, the intracellular chloride content of the cells was measured. The internal Cl^- ^concentration was clearly related to the external concentration in the medium. At low Cl^-^_e_, Cl^- ^was largely excluded from the cytoplasm. Increasing Cl^-^_e _lead to increasing Cl^-^_i _until a steady state was reached at 0.5 – 0.8 M Cl^-^_e_. At that concentration, the internal concentration was found to be about half the external concentration, leading to the first idea of chloride serving as a solute in *Halobacillus halophilus *[47] (Fig. 1). This hypothesis was questioned when later investigations revealed that chloride does not only support growth but also is essential for motility and endospore germination [48,49]. These observations pointed to a more global role of chloride in the physiology of *Halobacillus halophilus*. To address this question the effect of Cl^- ^on motility was analyzed. Biochemical and molecular data proved that transcription of *fliC*, encoding the structural protein of the flagellum, as well as flagellin production were strictly dependent on chloride and these studies gave rise to the hypothesis that chloride could serve as a newly discovered signal molecule in *Halobacillus halophilus *[50].

A comparison of the proteome of nitrate and chloride grown cells revealed 5 additional proteins that were specifically produced in the presence of chloride. Among those proteins, which were tentatively identified by BLAST searches, members of the general stress regulon, as described for *B. subtilis *[51,52], were found. One was SodA, a superoxide dismutase, another YvyD, a modulator of the sigma factor σ^L^. Furthermore, an N-acetylmuramoyl-L-alanine amidase that is essential in cell wall biosynthesis, a protein with unknown function and a protein (LuxS) that is essential in the production of autoinducer-2 or in the activated methyl cycle were found [50]. For the latter the proteome data were confirmed by additional studies, which not only revealed a chloride dependent production of LuxS, but also showed a clear influence of chloride on *luxS *transcription [53]. In sum, the data obtained from proteome analysis suggest the existence of a global regulatory network of processes that are dependent or at least are strongly influenced by chloride [54].

## The main compatible solutes in *Halobacillus halophilus *and their biosynthesis

Since Cl^- ^is indispensable for growth it has to influence a growth essential process. The nature of this process was unidentified for a long time. For a moderate halophile a definitely essential function is to measure external salinities and to adapt to changing osmolarities in the habitat. *Halobacillus halophilus*, as already mentioned, was isolated from salt marshes. The salt marshes represent an elevated, coastal area that is flooded during high tides with sea water that possesses an average salt concentration in the North Sea of about 3.5%. The salt is mainly NaCl. Upon evaporation and additional salt agglomeration by the sea wind the salt concentrations can rise up to 10% in the salt marshes, but drop almost to the level of freshwater after rainfall. The first hint that chloride could be involved in osmoregulation and the accumulation of compatible solutes came with investigations published by Roeßler and Müller in 2001. Transport studies with radiolabelled glycine betaine revealed a strict sodium and chloride dependence of glycine betaine uptake as response to osmotic upshock. Inhibitor studies suggested a primary transport system [55]. In the absence of glycine betaine, *Halobacillus halophilus *copes with the effect of external salt by accumulating of a cocktail of different compatible solutes, mainly amino acids such as glutamine, glutamate, proline and alanine but also of amino acid derivatives such as N^ε^-acetyl lysine, N^δ^-acetyl ornithine, glycine betaine and ectoine [56]. The obvious question that arose from these considerations was whether the accumulation of compatible solutes *via de novo *biosynthesis was also a chloride-dependent process and whether this was the key to an explanation for the chloride dependence of *Halobacillus halophilus*. To address this question intensive biochemical and physiological studies were done to solve the pathways for compatible solute biosynthesis, to solve their regulation and to clarify the role of chloride in these processes.

### The biosynthesis of glutamate and glutamine

For the biosynthesis of glutamate and glutamine three main enzymatic reactions are known in biology. The biosynthesis of glutamate can either be accomplished by the action of a glutamate synthase (GOGAT) or by the action of a glutamate dehydrogenase (GDH) while glutamine is synthesized by the action of a glutamine synthetase (GS).

**Figure 2:**
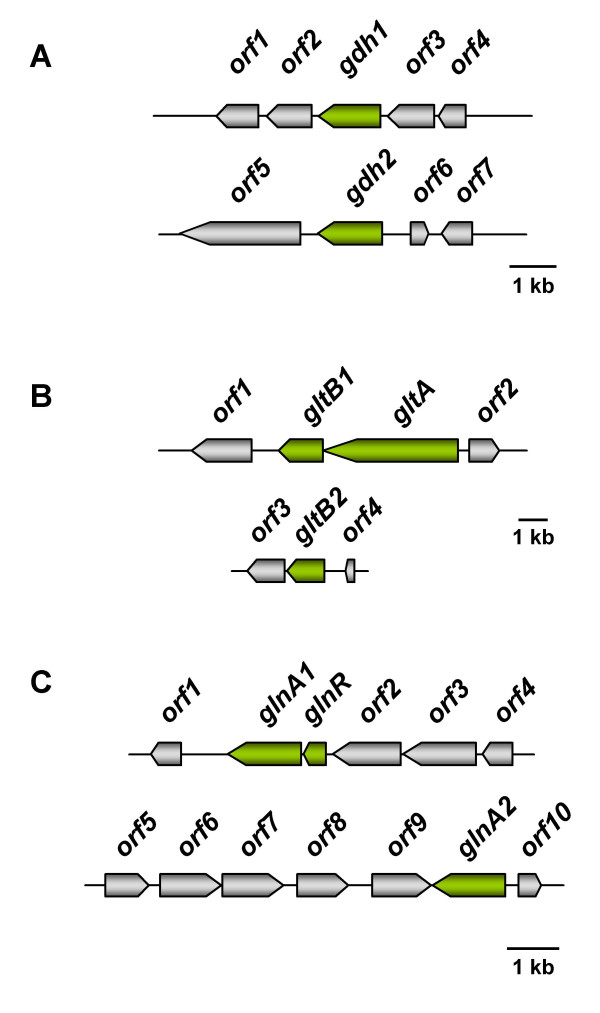
**Genetic organization of genes potentially involved in the biosynthesis of glutamine and glutamate in *Halobacillus halophilus***. **(A) **Glutamate dehydrogenase (*gdh*). Two ORFs encoding a putative glutamate dehydrogenase (*gdh1 *and *gdh2*) could be identified. *orf1*: L-asparaginase, *orf2*: pyridine nucleotide-disulfide oxidoreductase family protein (thioredoxin reductase), *orf3*: D-alanyl-D-alanine ligase A, *orf4*: negative regulator of genetic competence, *orf5*: unknown, *orf6*: enoyl-[acyl-carrier protein] reductase, *orf7*: unknown. **(B) **Glutamate synthase (*glt*). The glutamate synthase is a heterodimer, encoded by *gltA *(large subunit) and *gltB *(small subunit). In the genome of *Halobacillus halophilus *only one ORF could be identified encoding GltA whereas two ORFs were found encoding GltB (*gltB1 *and *gltB2*). *orf1*: catalase, *orf2*: L-aminopeptidase/D-esterase, *orf3*: dihydropyrimidine dehydrogenase, *orf4*: β-alanine synthase. **(C) **Glutamine synthetase (*glnA*). *orf1*: hypothetical conserved protein, *glnR*: transcriptional regulator, *orf2*: aluminum resistance protein, *orf3*: GTP-binding protein, *orf4*: spore formation protein, *orf5*: ABC transporter protein, *orf6*: oxidoreductase, *orf7*: putative dehydrogenase, *orf8*: conserved protein, *orf9*: conserved hypothetical protein/GTPase of unknown function, *orf10*: hypothetical protein.

Based on the genome sequence the situation in *Halobacillus halophilus *resembles that of *Bacillus subtilis *[57]. Two putative open reading frames encoding a glutamate dehydrogenase (*gdh1 *and *gdh2*) were found and only one open reading frame encoding the large subunit of a glutamate synthase (*gltA*), but two open reading frames each potentially encoding the small subunit of a glutamate synthase (*gltB1 *and *gltB2*) were identified [58] (Fig 2). However, transcriptional analyses did not reveal a salinity dependence of expression of *gdh1*, *gdh2 *or *gltA *although the glutamate concentration increased with increasing salinity. Furthermore, almost no glutamate dehydrogenase or glutamate synthase activity was detected. A clue to the origin of glutamate in *Halobacillus halophilus *came up the first time when *Halobacillus halophilus *cells were subjected to an osmotic upshock from 0.8 to 2.0 M NaCl rather than using preadapted cells. While the genes encoding the subunits for the glutamate synthase were not affected by the sudden increase of salinity, one of the putative glutamate dehydrogenase genes (*gdh1*) was induced and the mRNA level increased within 1.5 hours to about four-fold compared to the level before the upshock. It was therefore suggested that glutamate that is used as a compatible solute in *Halobacillus halophilus*, is produced by the catalytic activity of a glutamate dehydrogenase that converts one molecule of 2-oxoglutarate and one molecule of NH_4_^+ ^reductively to one molecule of glutamate [59]. Quantification of the transcripts of the second glutamate dehydrogenase gene (*gdh2*) showed that they were close to the detection limit which caused a rather high error probability and therefore no final conclusion on the regulation could be made. However, the presence of transcripts points to a physiological functionality. It is quite conceivable that the second glutamate dehydrogenase is involved in nitrogen metabolism rather than osmoregulation. Further, this function could be in a catabolic way as observed in *B. subtilis *or in an anabolic way under nitrogen excess as observed in *Escherichia coli*. A role in nitrogen metabolism was also suggested for the glutamate synthase.

Glutamine is synthesized by the action of a glutamine synthetase in *Halobacillus halophilus *[58]. In the genome two open reading frames could be identified each encoding a putative glutamine synthetase. While the first one (*glnA1*) is clustered with a gene (*glnR*) encoding a regulatory protein with very high similarity to the regulator GlnR of *B. subtilis *that is known to be essential in nitrogen metabolism, the second one (*glnA2*) lies solitary and is preceded by a putative promoter that shares very high similarity to the promoter of *B. subtilis *that is recognized by σ^B^, the general stress σ-factor. Analysis of mRNAs of cells cultivated at different salinities (0.4 – 3.0 M NaCl) revealed a strongly salinity-dependent expression of *glnA2 *with a maximal increase of transcripts of about 4-fold (compared to the value at 0.4 M NaCl) at 1.5 M NaCl or higher. Expression of *glnA1*, however, was not influenced by different salinities. The existence of two genes for the same enzyme can be regarded as a simple means for regulation that makes it feasible to separate and process completely different physiological demands: nitrogen-metabolism and osmoregulation.

A glutamine synthetase activity was readily detectable and it was apparent that this activity increased with increasing salinity of the medium [58]. Interestingly, maximal activity was not found at intermediate salt concentrations as expected from the mRNA data and the glutamate concentration measured in the cells, but was found at 2.5 M NaCl or higher. This observation does not necessarily reflect a contradiction, but rather underlines the role of glutamine, but also glutamate, that can easily be made of glutamine, as precursor molecules for other building blocks and finally compatible solutes like proline or ectoine at higher salinities, as discussed later.

Expression of *glnA2 *appeared to be affected only slightly, but significantly by the presence or absence of the anion chloride. This is perfectly in line with previous studies, where regulation on the transcriptional level was shown to be of minor importance in *Halobacillus halophilus *[50,53]. A more striking influence of chloride was found on the level of glutamine synthetase activity, which appeared to be strictly chloride dependent. The way how chloride can influence the activity of the glutamine synthetase is not elucidated yet, but principally two different modes of regulation are conceivable. First, and this is the easiest one, chloride could directly bind to the enzyme and influence its stability and/or conformational state. It is known from previous work that chloride is available within the cell [47] and putative basic residues to which chloride could bind were identified in the protein sequence of the *Halobacillus halophilus *glutamine synthetase. Second, the glutamine synthetase could be regulated by a regulatory protein that directly or indirectly senses the concentration of chloride. To clarify this question, future efforts have to be focused on the chloride dependence of purified glutamine synthetase and the question of how different anions can bind and influence the activity of the glutamine synthetase. However, the finding of a chloride dependent compatible solute production, for the first time, gave a rationale for the chloride dependence of *Halobacillus halophilus*, since growth in saline environments is only feasible with a functional active osmoregulation.

### The biosynthesis of proline

**Figure 3:**
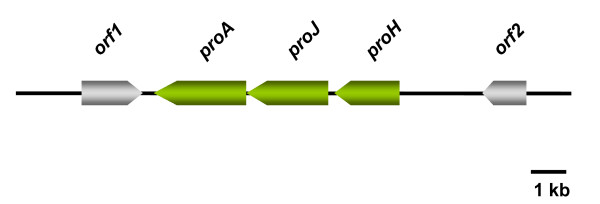
**The proline operon of *Halobacillus halophilus***. The proline operon of *Halobacillus halophilus *comprises 3 genes encoding a pyrroline-5-carboxylate reductase (*proH*), a glutamate 5-kinase (*proJ*) and a glutamate 5-semialdehyde dehydrogenase (*proA*). The gene products are sufficient to synthesize proline from glutamate **(Fig. 5)**.

Proline is accumulated in *Halobacillus halophilus *as a response to increasing salinity and even dominates over glutamine and glutamate as the major compatible solute at salinities of 2.0 M NaCl or higher [60]. The inspection of the genome revealed a cluster of three genes – *proH*, *proJ *and *proA*, which are organized in an operon – that encodes for putative proline biosynthesis enzymes (Fig. 3). The amount of transcript was found to be correlated with the salinity of the medium and was maximal at 2.5 M NaCl. At the same time proline concentrations were maximal at 3.0 M NaCl. The corresponding enzymes that are encoded by these genes comprise a putative pyrroline-5-carboxylate reductase (ProH), a putative glutamate 5-kinase (ProJ) and a putative glutamate 5-semialdehyde dehydrogenase (ProA). Therefore, the three enzymes are sufficient to synthesize proline from glutamate.

Unlike *B. subtilis *[57], no other genes were found that encode for potential proline biosynthesis enzymes and, therefore, the genes/proteins identified in *Halobacillus halophilus *have to respond to different stimuli, such as low proline-content within the cell and high salinity. Analyses of *proHJA *transcript levels, where the molecular answer to an increase in salinity was addressed, revealed an almost immediate increase of mRNA after an osmotic upshock that reached a maximum already 1.5 hours after the upshock [60]. At the same time the proline concentration increased and reached a maximum after 6 hours. This result demonstrated that salinity triggers the production of proline, but left open the question on the actual signal that leads to the increase in proline production preferentially at high salinities. A clue what the actual signal could be came up when the same experiment was performed with Na-glutamate instead of NaCl. Interestingly, glutamate also led to an accumulation of proline, but in contrast to NaCl the addition of Na-glutamate dramatically increased the *proHJA *mRNA concentration. The stimulating effect of glutamate on the transcription of the *pro *genes was subsequently titrated and it was found that a minimal concentration of 0.2 M glutamate is sufficient to stimulate *pro *gene transcription [60]. Following these data it was speculated that not (only) NaCl is the initial signal that triggers the production of proline, but the internal glutamate concentration, that increases with increasing salinity. Glutamate is therefore regarded as a "second messenger" in *Halobacillus halophilus *beside of being a compatible solute.

Based on these data it is not surprising that glutamate is able to substitute chloride in growing cells [61]. Glutamate was shown to be taken up and accumulated in a chloride-independent manner. Within the cell it then serves as the major compatible solute and as a "second messenger" leading to enforced transcription of several genes involved in osmoregulation, for example such genes as *glnA2 *or *proHJA*. Moreover, it can be assumed that transcription of additional genes is also stimulated by the presence of glutamate and, therefore, growth is allowed in the absence of chloride. However, it should be kept in mind, that 1 M Na-glutamate is artificial and not encountered in the ecosystem. Interestingly, glutamate cannot short-circuit the Cl^- ^– regulon in general. The biosynthesis of flagellin and therefore motility cannot be achieved in the absence of chloride but presence of glutamate.

### The biosynthesis of ectoine

**Figure 4:**
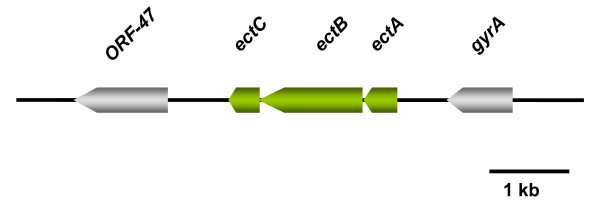
**The ectoine operon of *Halobacillus halophilus***. The ectoine operon of *Halobacillus halophilus *comprises 3 genes encoding a diaminobutyric acid acetyltransferase (*ectA*), a diaminobutyrate-2-oxoglutarate transaminase (*ectB*) and an ectoine synthase (*ectC*). The reaction sequence that is catalyzed by the gene products is shown in **Fig. 5**.

Ectoine is a compatible solute in *Halobacillus halophilus *that is mainly produced at high salinities, but in contrast to other organisms ectoine is not the dominant solute at high salinities [62]. The dominant solute at high salinities is proline rather than ectoine, as mentioned above, although the salinity dependent concentration pattern in steady state cultures resembles that of proline. The biosynthetic genes (*ectABC*) for the production of ectoine from aspartate semialdehyde were identified and were shown to form an operon (Fig. 4). In studies that addressed the regulation of ectoine biosynthesis in steady state cultures, a clear correlation between the *ectABC *mRNA levels and the ectoine concentration was found. While the transcripts and the ectoine concentrations were low at low salinities (0.4 – 1.5 M NaCl), they increased at elevated salinities (2.0 – 3.0 M NaCl). Therefore, ectoine production is part of the osmoregulation. The first clear discrimination of the roles of proline and ectoine became obvious when the solute concentration were determined during growth. While proline concentrations dramatically decreased when the cells entered the stationary phase ectoine concentrations increased and became maximal in the late stationary growth phase. This result nicely fits to the report of Calderón and co-workers who found, using *E. coli *as a heterologous host, maximal transcription of the *Chromohalobacter salexigens ect *promoter fused to the *lacZ *gene (*P*_*ect*_*-lacZ*) in the early stationary growth-phase [63]. This observation hints to a novel, additional layer of regulation and it can be assumed that ectoine production in the late stationary phase reflects an answer to the stress situation that the cells experience in stationary cultures. To resolve the time dependent kinetics of ectoine production cells were subjected to an osmotic upshock from 0.8 to 2.0 M NaCl and the biosynthesis of ectoine was measured at the levels of transcription, translation and solute accumulation [62]. Transcripts were readily detectable already at time-point 0 h, but increased dramatically with time and reached a maximum not before 3 hours after upshock. Most important, expression of *ect*-genes was preceded by expression of genes responsible for glutamine, glutamate or proline biosynthesis. The signal leading to *ect *gene transcription is therefore assumed to be an indirect one mediated by one or more yet to be identified factors rather than by the presence of the osmolyte. The production of the ectoine synthase EctC nicely corresponds to the increase of *ectC *transcript. Both were found to increase 2-fold after 4 hours after upshock. Surprisingly, 4 hours after upshock the EctC content again decreased with time and the level reached a value only slightly above the value at the beginning, although the external stress was still present. This decrease, however, was not reflected in the ectoine concentration, which steadily increased and reached a maximum after 18 hours after upshock [62]. Again, this demonstrates a great delay in accumulation compared to proline that reached its maximum already 6 hours after upshock and hints to a role of ectoine not only in the immediate response to osmotic upshock but to a function as a more general protectant in the cell. This idea is corroborated by the results of different groups that showed that ectoine is able to function as a very powerful stabilizing agent of whole cells or enzymes against a number of stresses like thermal denaturation, cryo – damage or UV radiation [64-67]. An explanation for the only temporary increase of protein would be that the reaction catalyzed by this enzyme is not the rate-limiting step in ectoine biosynthesis. An increase in enzyme concentration is not necessary and translation therefore reduced. It is also conceivable that the ectoine synthase is regulated on the activity level by the increase of chloride within the cell and, therefore, makes an increase of EctC almost unnecessary.

## Sequence of events in osmoadaptation in *Halobacillus halophilus*

### Salinity-dependent osmoregulation

**Figure 5:**
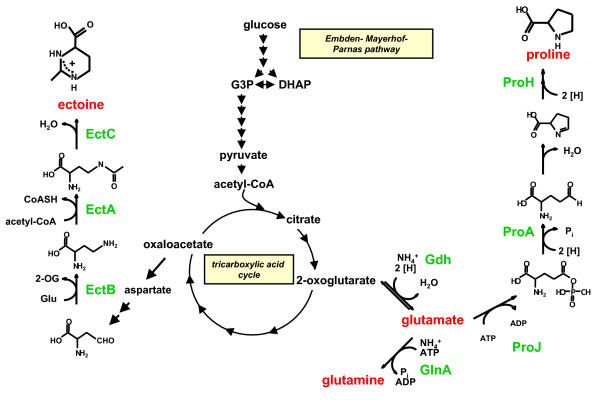
Proposed biochemical pathways of the main compatible solutes in *Halobacillus halophilus*.

**Figure 6:**
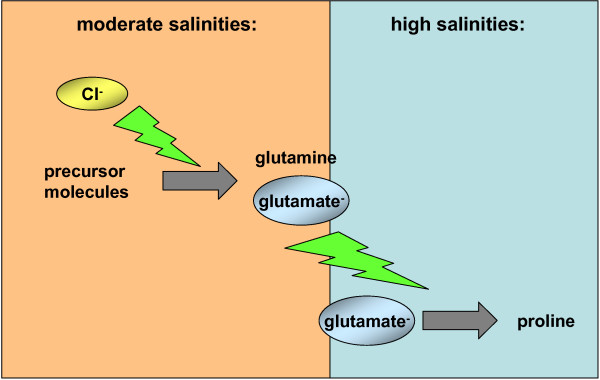
***Halobacillus halophilus *switches its osmolyte strategy from glutamine/glutamate at moderate salinities to proline at high salinities**. Upon an increase of salinity of the growth medium the intracellular glutamine/glutamate pool increases. When the glutamate concentration reaches a critical concentration it effectively triggers the transcription of the *pro*-genes and, therefore, proline production.

*Halobacillus halophilus *synthesizes different solutes and their biosynthetic routes (Fig. 5) and their regulation have been identified. Based on our studies the following model for long-term osmoadaptation in steady state cells of the moderate halophile *Halobacillus halophilus *was proposed. Challenged by low extracellular water activity, caused by elevated NaCl concentrations, *Halobacillus halophilus *maintains a rather high internal Cl^- ^concentration in the molar range but additionally accumulates compatible solutes. At intermediate salinities (1.0 – 1.5 M NaCl) glutamate and glutamine are accumulated in correspondence to the external salinity. Upon a further increase of salinity the glutamate-pool reaches a critical threshold value that now effectively triggers the transcription of the *pro *– genes and therefore the production of proline. This event initiates the switch to proline as the dominant compatible solute at higher salinities (2.0 – 3.0 M NaCl) (Fig. 6). It is noteworthy to mention that the production of glutamine/glutamate as well as the production of proline is a directly or indirectly chloride-dependent process. The physiological reason for this change in osmolyte strategy is still an open question in the field of osmoregulation, but seems to be common in the domain of *Bacteria *and *Archaea *[68-70]. A reasonable explanation for the preferential use of compatible solutes could be due to different properties in protecting cellular compounds from different external influences like temperature or salinity [71]. Rabbit muscle lactate dehydrogenase, for example, is more efficiently protected by hydroxyectoine than by its precursor *N*^γ^-acetyldiaminobutyrate when incubated at 55°C [64]. Furthermore, *E. coli *glutamyl-tRNA treated with urea was more stable in the presence of D- or L-glutamate than in the presence of sorbitol, trimethylamine oxide, or inositol [72]. The exchange of compatible solutes at higher salinities could also be due to the toxic effect of potassium that is accumulated by Gram-negative and Gram-positive bacteria as a first response to increasing salinities as described above. In this regard glutamate serves as the counter-ion for K^+^. Since the accumulation of potassium is limited due to its toxic effect, K-glutamate has to be replaced by other solutes like proline that can be accumulated to higher concentrations without disturbing the cell metabolism.

### Time-dependent (temporal) response

**Figure 7:**
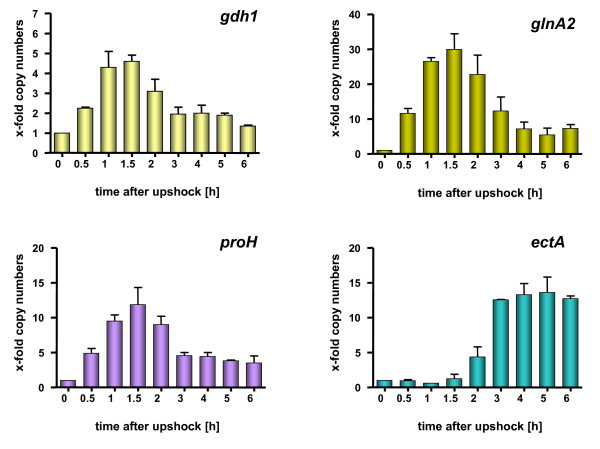
**Transcription of *ect-*genes is delayed after an osmotic shock compared to other genes involved in the production of compatible solutes**. *Halobacillus halophilus *cells were cultivated in mineral medium in the presence of 0.8 M NaCl to an OD_578 _of about 0.8. After harvesting and washing the cells were directly resuspended in mineral medium containing 2.0 M NaCl to an OD_578 _of about 9. The cell suspension was incubated on a rotary shaker at 30°C and samples were taken at the time points indicated. From these samples RNA was prepared and transcribed into cDNA which was further used as template in a quantitative real-time PCR reaction using specific primer pairs for *gdh1*, *glnA2*, *proH *and *ectA*, respectively. The values given represent the ratio between the value at time point X and the value at 0 hours. Measurements were done in duplicate with 2 independent parallels [62].

For cells that were subjected to a sudden increase in salinity, the following time-dependent processes were derived: upon osmotic upshock chloride ions are accumulated (by (a) so far unknown mechanism(s)) and consequently the chloride concentration increases within the cells to about half the concentration found in the medium. Whether this process is accompanied by the uptake of K^+ ^as it was proposed for *E. coli *[16] is unknown but likely. Since the final chloride concentration is too low to support full osmoprotection, compatible solutes have to be accumulated additionally. In the presence of glycine betaine, chloride-dependent uptake of this compatible solute is preferred (at least partly) over *de novo *synthesis of other compatible solutes [55]; Saum, Koller, Santos, Müller unpublished data]. In the absence of glycine betaine, the anion chloride triggers the production of the compatible solutes glutamine and glutamate to counterbalance the low water activity of the medium [58]. The accumulation of glutamate in turn triggers effectively the transcription of the proline genes and leads to the accumulation of proline [60]. It should be mentioned that glutamate is not only the trigger but also the precursor for the synthesis of proline. This sequence of events is reflected by the time-dependence of expression of key genes of compatible solute biosynthesis (Fig. 7).

### Adaptations in stationary growth-phase

Cells, however, that were situated for a prolonged time at high cell densities replace proline (at least in part) by ectoine [62]. The ectoine biosynthesis and final concentration thereby depend on the salinity of the medium and the availability of chloride. Again, glutamate is crucial and serves as a NH_2_-donor in this pathway (Fig. 5). The mode of how *Halobacillus halophilus *cells sense the density of the culture is currently not known, but could be accomplished for example *via *quorum sensing. *Halobacillus halophilus *produces autoinducers of the furanone-type (autoinducer 2 [AI-2]) that accumulate in stationary phase cultures (Saum, Winzer, Müller unpublished data). Moreover, the transcription and translation of the key-enzyme for AI-2 production was shown to be chloride dependent as already mentioned above.

## The chloride regulon of *Halobacillus halophilus*

**Figure 8:**
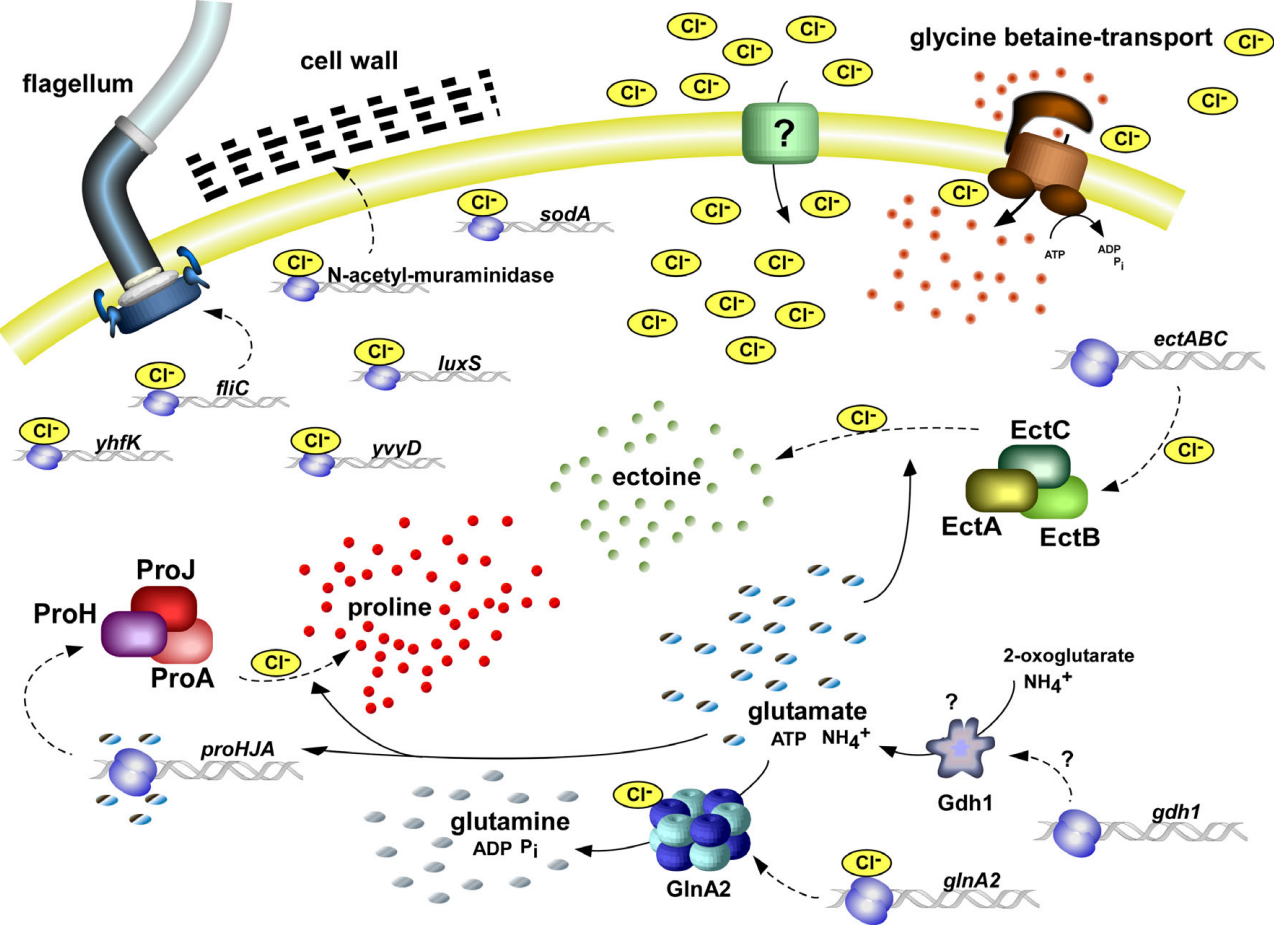
**The chloride regulon of *Halobacillus halophilus***. For further explanations see text.

The finding that chloride is essential for osmoprotection and the accumulation of the compatible solutes glutamine, glutamate, proline and ectoine extended the model of a chloride dependent regulatory network in *Halobacillus halophilus*. It was shown that chloride affects the accumulation of these compatible solutes very dramatically and acts as a regulator on different levels, namely transcription, translation, but also enzyme activity (Fig. 8). Based on these results, a reasonable explanation for the essential role of chloride in *Halobacillus halophilus *was given for the first time.

Concomitantly with the identification of the growth-essential process of osmoregulation as a chloride dependent process, the question about the molecular basis for chloride as a regulator molecule in this process comes up. In this context one has to keep in mind that before solutes are accumulated as a response to hyperosmolarity the cell has to sense osmolarity somehow. The process of sensing osmolarity is generally only poorly understood and almost not investigated in moderate halophiles. Studies with the halotolerant model organism *E. coli *lead to the assumption that the initial signal that is sensed after an osmotic upshock is the reduced turgor pressure that establishes due to a loss of water. This reduced turgor leads to an influx of K^+ ^within seconds *via *the potassium transporters Kup, Trk, Kdp [73,74] but also activates the KdpD-KdpE regulatory system which in turn regulates the expression of the *kdpFABC *operon encoding the Kdp transport system. The KdpD-KdpE system is a classical, bacterial regulatory two-component system with KdpD being the membrane-bound sensor kinase and KdpE being the response regulator. The actual mode of sensing the turgor pressure that leads to the autophosphorylation of KdpD is still a matter of debate, but was postulated to be due to alterations of the transmembrane helix-helix interactions, but additionally seems to be influenced by the K^+ ^concentration [75,76,29].

Contrary to this model, the osmosensing two-component system MtrB-MtrA of *Corynebacterium glutamicum *does not respond to a reduced turgor pressure after osmotic upshock. Instead, the addition of different chemical compounds, like sugars, amino acids or polyethylene glycols seems to trigger the autophosphorylation activity of MtrB. The proposed explanation for this is the change in the hydration state of the protein caused by the solutes [77]. In *Halobacillus halophilus *two-component systems are present, but none of them could definitely be assigned to be of the KdpDE or the MtrBA type. However, the actual signal that is recognized as well as the mechanism might be different in different organisms.

However, not only two-component systems may serve as potential osmo-sensors, but also transporter proteins. BetP of *C. glutamicum *or *E. coli *is a Na^+^/glycine betaine symporter of the betaine-carnitine-choline transporter family (BCCT) that is strongly regulated by the osmolality of the medium. The regulatory domain was shown to be located in the C-terminus that extends into the cytoplasm. Due to its basic characteristics it is believed to interact with the cytoplasmic membrane, but loses its interaction upon K^+ ^accumulation after osmotic upshock. The loss of interaction in turn leads to a conformational change within the protein and to an increase of specificity for its substrate glycine betaine [78-80].

A similar mechanism of "sensing" the surface charge of the membrane was proposed for the ATP-hydrolyzing ATP-transporter OpuA of *Lactococcus lactis*. OpuA is a high-affinity transporter for glycine betaine that effectively can be switched on or off by osmotic upshift or downshift, respectively [81]. The molecular sensors with which the osmotic strength can be measured were identified to be the C-terminal CBS domains of the ATP-hydrolyzing subunits. They possess a very anionic character and were therefore postulated to be influenced by the negatively charged membrane. Similar to BetP the change of the ionic strength could result in a conformational change and therefore activation of the transporter [82].

In contrast to the enteric bacteria, like *E. coli *for example, or the Gram-positive bacterium *C. glutamicum*, that have to be able to respond to fluctuating osmolality rather than salinity, the major osmolyte *Halobacillus halophilus *is confronted with, is NaCl. It would therefore be an attractive explanation that *Halobacillus halophilus *senses changing salinities by sensing changing chloride concentrations. The actual mechanism is unclear to date, but several models are conceivable: (i) Chloride could be measured via an extracellular receptor. The binding of substrate would then trigger a signal transduction cascade into the cell similar to a two component system. The disadvantage of this model would be that it cannot explain how the intracellular osmotic state is measured. (ii) Another concept could be based on the idea that changes in salinity could be measured *via *a chloride transport process into the cell. The import could be measured by a specific transporter or by an associated protein which then leads to a signal transduction cascade and finally to a cellular response. A similar model was already proposed for the moderately halophilic bacterium *Halomonas elongata *where the transport of the compatible solute ectoine is thought to be the osmosensor [83]. (iii) A chloride-binding regulator in the cytoplasm could directly interact with the anion and could, depending on the chloride concentration, trigger cellular responses. One putative candidate for such a function is the regulator protein YvyD that was identified in chloride-but not in nitrate-grown *Halobacillus halophilus *cells [50]. The latter two models have the advantage that they provide a possible mechanism, with which changes in the external salinity – that are reflected in the intracellular chloride concentrations – can be measured. However, the fact that very different processes are affected by the presence or absence of chloride in *Halobacillus halophilus *demands the presence of a global regulatory system. The crucial point of these concepts is that they cannot be explained by the presence of only one regulatory protein, since no regulator is known to date that is able to regulate on such different targets as transcription, translation and enzyme activity at the same time. In *Halobacillus halophilus *no direct evidence was found so far, with exception of YvyD, that a multi component regulatory network of chloride dependent regulators exists.

## Physico-chemical effects of (an)ions on biological systems

**Figure 9:**
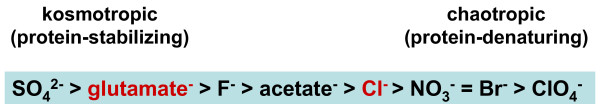
**The Hofmeister series**. The Hofmeister series describes the potential of ions (here anions) to stabilize or denature proteins in solution. In this series (modified to the original Hofmeister series [84]) glutamate^- ^possesses a more stabilizing effect than Cl^- ^which is used by *Halobacillus halophilus *for regulatory purposes.

The regulating mechanisms listed above require specific ion-target interactions and are functional already at very low ion concentrations. The functionality and specificity is based mainly on the chemical properties of the specific ion. But with increasing ion concentrations the physical effects of the salt solution becomes more pronounced and unspecific interactions (direct or indirect) become important. Among the effects that are caused by high salt concentrations are (i) the depletion of water molecules, (ii) a change in water activity, (iii) osmotic activity or (iv) hydrophobic interactions. A conceivable model for sensing osmolarity, therefore, would also imply the measurement of such physical parameters by cellular compounds. The effect of inorganic ions on biological matter was investigated already at the end of the 19^th ^century. Franz Hofmeister detected that especially anions have the capacity to precipitate proteins (globuline) out of solutions. The capacity to do so is different for different anions. Based on this observation he was able to rank the anions in a series that became known as the "Hofmeister series" (Fig. 9). In this series sulfate had the strongest influence on precipitation followed by phosphate, acetate, citrate, tartrate, bicarbonate, chromate, chloride, nitrate and chlorate [84]. Centuries later the exact molecular basis for the Hofmeister series is still enigmatic, but it becomes more and more obvious that it is due to direct ion-macromolecule interactions and interactions of ions with water molecules in the first hydration shell of the macromolecule. Thereby, kosmotropic ions are differentiated from chaotropic ions. Kosmotropic ions are strongly hydrated, act stabilizing on macromolecules and show a salting-out effect. In contrast, the chaotropic ions directly interact with macromolecular surfaces and destabilize folded proteins. They exhibit a salting-in behavior [for review and further reading see [85] [86]. The relevance of the Hofmeister series in biological systems was demonstrated by several studies that focused on enzyme activity [87-89], on protein stability [90,91], on protein-protein interactions [92,93] or on bacterial growth [94,95].

Based on the Hofmeister series and the effects of anions on proteins or whole cells, a different concept can be proposed for *Halobacillus halophilus *to measure osmolarity. *Halobacillus halophilus *is a moderately halophilic organism that has adapted to saline environments. Since chloride is not excluded from the cytoplasm but rather accumulates, depending on the external salinity, to molar concentrations within the cell [47], it is principally thinkable that the physical effects caused by the presence of chloride is of regulatory importance. A prerequisite for the usage of such a strategy is the evolutionary development of proteins that are functional in the presence of such high ion concentrations. As a consequence such enzymes might be dependent on the presence of minimal Cl^- ^concentrations for optimal functionality. Examples for such a behavior were already found in *Halobacillus halophilus *in the case of the glutamine synthetase that demands an optimal salinity of 1.5 M NaCl [58]. Such an adaptation to high salinities is already observed in extremely halophilic organisms such as members of the *Halobacteriaceae *or *Salinibacter ruber *and it would be in good correlation with the identification of 7 positively charged residues in the deduced protein sequence of GlnA2 – the postulated chloride dependent glutamine synthetase – at which chloride could bind [58]. The Hofmeister series not only offers an explanation of the regulatory influence of chloride on cell metabolism in *Halobacillus halophilus *but also offers an explanation to the regulatory influence of glutamate in the process of *pro*-gene transcription. Under physiological pH glutamic acid is deprotonated to the anion glutamate^- ^that accumulates within the cell to molar concentrations. Since too high concentrations of such anions are inhibitory for cell metabolism, the cell has to substitute glutamate^- ^by an electroneutral compatible solute like proline. The same mechanism was previously proposed for *E. coli *that substitutes glutamate^- ^by trehalose [16]. Again, the cell has to "sense" the concentration of an anion (in this case glutamate^-^). For *Halobacillus halophilus *it was shown that a minimal external glutamate concentration of 0.2 M is sufficient to stimulate *pro *gene transcription very effectively [60]. Following these data it was speculated that not (only) NaCl is the initial signal that triggers the production of proline, but also the internal glutamate concentration, that increases with increasing salinity. Glutamate is therefore regarded as a "second messenger" in *Halobacillus halophilus *beside of being a compatible solute. This idea was nicely corroborated by studies done in the group of Prof. Gralla that demonstrate the potential of glutamate as an activator or inhibitor of transcription [96,97]. In sum, based on the Hofmeister series a very sophisticated model of regulatory sequences can be proposed for the osmoregulation in *Halobacillus halophilus *that is solely based on physico-chemical properties of anions.

## An intermediate strategy for a moderate halophile

A still open question is why *Halobacillus halophilus *additionally accumulates compatible solutes to molar concentrations although there are high concentrations of chloride present in the cell (Fig. 1). The advantage of such a hybrid strategy (salt-in and compatible solute in) lies at hand. Strictly halophilic archaea are dependent on molar concentrations of NaCl in their environment and exclusively use the salt-in strategy to counterbalance the low water activity. Although single members of the haloarchaea are known to be able to grow over a broad range of salinity (*H. volcanii *for example), they still depend on minimum NaCl concentration of 1.5 M or higher to grow. This dependence is due to a very pronounced adaptation of the whole enzymatic machinery on high salt concentrations. Such a radical adaptation would be counterproductive for a moderate halophile like *Halobacillus halophilus *thriving in habitats where salinities temporarily can reach molar concentrations but also can fall to freshwater concentrations after rainfalls. Instead, an only modest adaptation to elevated salinities on the protein level would be advantageous. It could help the cell to tolerate a sudden or dramatic increase of salinity that concomitantly would result at least in a temporary increase of salt concentration in the cytoplasm, easier, since enzymes are still active. However, such an adaptation on the enzyme level requires the presence of a minimal salinity outside and within the cell for optimal operation and could therefore explain the chloride concentrations measured in *Halobacillus halophilus*. The nature of the cation that balances the negative charge of chloride is not yet known, but could be K^+^. An only modest adaptation to elevated salinities within the cell also explains the need for alternative osmoprotectants at high external salinities. Therefore, *Halobacillus halophilus *has to accumulate compatible solutes such as glycine betaine, glutamate, glutamine, proline or ectoine in addition.

In summary, a dual strategy in osmoprotection is able to explain the broad range of tolerated salt concentrations that is common to all moderately halophilic organisms. In the context of this model *Halobacillus halophilus *represents an intermediate in respect to its demands of NaCl between the non halophilic and the extreme halophilic organisms. It is therefore conceivable that this organism employs an intermediate strategy of salt-in-cytoplasm and the use of compatible solutes.

## Future perspectives

The model of *Halobacillus halophilus *as an organism that uses an intermediate strategy for osmoregulation and physico-chemical properties of anions to regulate the processes of compatible solute accumulation opens a whole new field of regulatory mechanisms to study. Future investigations will have to show for example whether chloride directly influences the activity of the glutamine synthetase or if the stimulation of activity is mediated by a so far unknown factor. Further, the role of glutamate will have to be investigated in more detail. For this purpose *in vitro *experiments have to be established to study its effect on transcription. Also the role of ectoine will be an issue for further investigations. Can ectoine production also be triggered by other stimuli than salinity? And what then happens in an ectoine-deficient mutant? The answer at least to the last question demands the availability of a genetic system that hopefully will be at hand soon.
